# Non-Hodgkin's Lymphoma in Pregnancy: A Report of a Rare Case

**DOI:** 10.7759/cureus.91156

**Published:** 2025-08-28

**Authors:** Apoorva B Patil, Barathi Raja Kuppusami, Samir Ahmad

**Affiliations:** 1 General Surgery, Sree Balaji Medical College and Hospital, Chennai, IND

**Keywords:** lymphoma, non-hodgkin's lymphoma, pregnancy malignancies, r-chop chemotherapy, slow-growing tumors

## Abstract

Non-Hodgkin's lymphoma is a rare diagnosis observed in pregnant women. It can present as B-cell and T-cell lymphomas. It can be a diagnostic challenge in pregnant women, due to overlapping clinical features such as weight loss and fatigue, similar to pregnancy. It can be managed either by immediate intervention in aggressive cases or after postpartum if indolent. We present the case of a 30-year-old woman at 32 weeks of gestation, presenting with a painless progressive swelling over the left side of her neck. It was later diagnosed as non-Hodgkin's lymphoma and successfully managed by R-CHOP chemotherapy (rituximab, cyclophosphamide, doxorubicin, vincristine, and prednisolone).

## Introduction

Lymphomas are a heterogeneous group of lymphoid malignancies that rarely occur during pregnancy, with an estimated incidence of one in 6,000 pregnancies [1]. These are broadly categorized into Hodgkin's and non-Hodgkin's lymphoma. Non-Hodgkin's lymphoma encompasses a broad range of diseases, spanning from slow-growing to highly aggressive cancers [2]. Although non-Hodgkin's lymphoma is less commonly encountered in women of reproductive age compared to Hodgkin's lymphoma, its aggressive subtypes, such as diffuse large B-cell lymphoma, manifest during pregnancy. An overall incidence of non-Hodgkin's lymphoma has been reported as 18.7/100,000 [3]. Immunosuppression, ultraviolet radiation, viruses and other pathogens (Epstein-Barr Virus (EBV), human T-cell lymphotropic virus (HTLV), human herpesvirus 8 (HHV-8), hepatitis C, simian virus 40 (SV40), and *Helicobacter pylori*), autoimmune diseases, chronic inflammatory disorders, and occupational exposure are marked risk factors for non-Hodgkin's lymphoma [4]. The majority of non-Hodgkin lymphoma is caused by B-lymphocytes, and only a few have been associated with T-lymphocytes and natural killer cells [5,6]. Pregnancy introduces significant physiological and immunological changes that can obscure or mimic the presentation of lymphoma, such as fatigue, anemia, or lymphadenopathy. Diagnostic imaging and histopathological confirmation may be delayed due to fetal safety concerns, potentially impacting maternal prognosis. Therapeutic decisions must carefully weigh the urgency of treatment against gestational age and fetal risk, necessitating a multidisciplinary approach [7]. We present a rare case of non-Hodgkin's lymphoma diagnosed during the third trimester of pregnancy, managed with prompt histological confirmation and systemic chemotherapy, resulting in favorable outcomes for both the mother and the fetus. This case highlights the importance of early suspicion, individualized management, and collaborative care in optimizing outcomes in such complex clinical scenarios.

## Case presentation

A 30-year-old woman, G2P1, at 32 weeks of gestation, presented with a progressively enlarging, painless swelling in the left side of her neck for six weeks. The swelling was insidious in onset and progressively increasing in size. She also reported mild fatigue and intermittent low-grade fever but denied any significant weight loss, night sweats, or cough. Her antenatal history was otherwise unremarkable, and routine obstetric scans had shown normal fetal growth and well-being. She had no prior history of tuberculosis, autoimmune diseases, or malignancy, and there was no significant family history. On physical examination, the patient was afebrile, with stable vital signs. Local examination revealed multiple, firm, non-tender, mobile lymph nodes in the left cervical region (levels 2 and 3), the largest measuring 4.0 × 3.0 cm. An extensive differential workup was limited antenatally due to fetal safety. Basic laboratory tests, lactate dehydrogenase (LDH), erythrocyte sedimentation rate (ESR), and neck ultrasound with fine needle aspiration cytology (FNAC) were performed to exclude common causes such as tuberculosis and reactive lymphadenopathy. Definitive excision biopsy with immunohistochemistry postpartum confirmed diffuse large B-cell lymphoma and ruled out other differentials.

**Figure 1:**
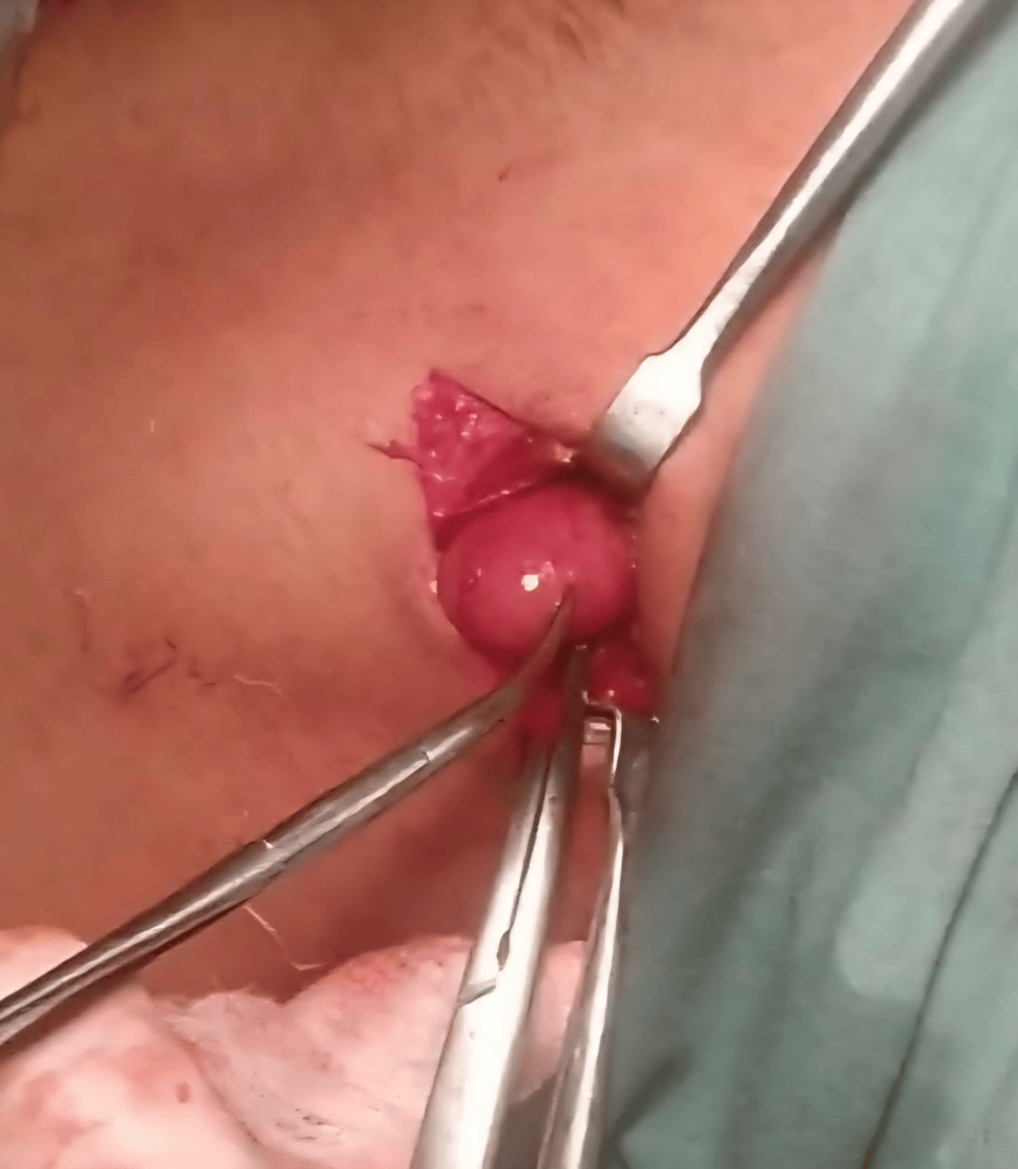
Excisional biopsy of an enlarged level 2a cervical lymph node for suspected non-Hodgkin's lymphoma

There were no palpable axillary or inguinal lymph nodes. Abdominal examination showed a gravid uterus consistent with 32 weeks of gestation, with cephalic presentation and regular fetal movements. Cardiotocography showed a reactive fetal heart pattern. Initial laboratory investigations revealed mild anemia (hemoglobin: 10.2 g/dL), elevated lactate dehydrogenase (LDH: 640 U/L), and a normal leucocyte count. Liver and renal function tests were within normal limits. Erythrocyte sedimentation rate was elevated at 52 mm/hour. Obstetric ultrasound showed a single live intrauterine fetus in cephalic presentation with appropriate growth parameters and normal amniotic fluid index. Due to gestational age and concern for fetal safety, a high-resolution ultrasound of the neck was performed, revealing multiple enlarged, hypoechoic lymph nodes with loss of fatty hilum. Fine needle aspiration cytology (FNAC) was done for the patient, and it was inconclusive. Excision and biopsy were planned for the patient post-delivery (Figure 1).

The laboratory results of the patient are shown in Table 1.

**Table 1:** Blood test results of the pregnant patient diagnosed with non-Hodgkin's lymphoma g/dL: grams per deciliter, μL: microliter, %: percent, U/L: unit/liter, mm/hour: millimeters per hour WBC: white blood cell, LDH: lactate dehydrogenase, ESR: erythrocyte sedimentation rate

Laboratory parameter	Patient value	Normal value
Hemoglobin (g/dL)	8.2	12-16
WBC count (cells/μL)	18,200	4,500-11,000
Lymphocytes (%)	56	20-40
Platelets (cells/μL)	95,000	150,000-450,000
LDH (U/L)	920	135-275
ESR (mm/hr)	85	0-20

Given the late third trimester, absence of aggressive systemic symptoms, and stable fetal and maternal conditions, chemotherapy was deferred until delivery. The patient was closely monitored until 36 weeks, at which point she underwent an elective lower segment cesarean section (LSCS) under spinal anesthesia. A healthy female infant weighing 2.8 kg was delivered with APGAR scores of 9 and 10 at one and five minutes, respectively. Postpartum recovery was uneventful. One week after the delivery, the patient underwent an excision biopsy of the cervical lymph node under local anesthesia. Histopathology revealed diffuse effacement of nodal architecture with sheets of large atypical lymphoid cells, and immunohistochemistry was positive for CD20, CD10, and BCL6, with a high Ki-67 index, consistent with diffuse large B-cell (germinal center B-cell-like (GCB)) subtype. Subsequent contrast-enhanced computed tomography (CT) imaging of the neck, chest, abdomen, and pelvis revealed stage 2 disease involving cervical and mediastinal lymph nodes. Bone marrow biopsy was negative. The patient was started on R-CHOP chemotherapy (rituximab, cyclophosphamide, doxorubicin, vincristine, and prednisolone). At present, she has completed two cycles of chemotherapy and is tolerating well.

## Discussion

Non-Hodgkin's lymphoma encompasses a heterogeneous group of malignancies, commonly arising from lymphocytes. These malignancies widely vary in their clinical behavior and aggressiveness. It can be a diagnostic and therapeutic challenge when presented in pregnant women, attributed to physiological challenges during pregnancy [8]. In pregnant women, lymph node biopsy is considered comparatively safe. Staging can be carried out by ultrasound and magnetic resonance imaging, which can minimize fetal radiation exposure as compared to positron emission tomography (PET) scans and computed tomography [9]. This patient presented at 32 weeks of gestation, which is considered safe in terms of the risk of congenital malformations in the fetus as compared to the earlier trimesters of pregnancy. Therapeutic intervention can be based on symptomatic presentation; immediate intervention is required only in aggressive tumors, and treatment can be deferred in cases with indolent tumors until delivery [9,10]. There was an indolent presentation with minimal symptoms in this patient, and hence, the treatment was deferred until her delivery. The patient delivered a healthy 2.8 kg female infant at 36 weeks and was initiated R-CHOP therapy regimen thereafter, which was well-tolerated until two cycles till the date of submission of this article.

## Conclusions

Non-Hodgkin's lymphoma diagnosis can be challenging in pregnant women. It requires multidisciplinary management involving oncologists, neonatologists, obstetricians, and hematologists for optimal outcomes. Therapy can be initiated in aggressive cases with multi-agent regimens such as R-CHOP with postponement until delivery in non-aggressive cases.
